# Idiosyncratic Characteristics of Postural Sway in Normal and Perturbed Standing

**DOI:** 10.3389/fnhum.2021.660470

**Published:** 2021-05-17

**Authors:** Tania E. Sakanaka, Martin Lakie, Raymond F. Reynolds

**Affiliations:** ^1^School of Sport, Exercise & Rehabilitation Sciences, University of Birmingham, Birmingham, United Kingdom; ^2^Faculty of Medical Sciences, State University of Campinas, Campinas, Brazil

**Keywords:** balance, human standing, postural sway, sway velocity, ICC, consistency

## Abstract

**Objective:**

Are people with a characteristically large physiological sway rendered particularly unstable when standing on a moving surface? Is postural sway in standing individuals idiosyncratic? In this study, we examine postural sway in individuals standing normally, and when subtle continuous sinusoidal disturbances are applied to their support platform. We calculate consistency between conditions to verify if sway can be considered characteristic of each individual. We also correlate two different aspects of participants’ responses to disturbance; their sway velocity and their regulation of body orientation.

**Methods:**

Nineteen healthy adults (age 29.2 ± 3.2 years) stood freely on footplates coaxially aligned with their ankles and attached to a motorized platform. They had their eyes closed, and hips and knees locked with a light wooden board attached to their body. Participants either stood quietly on a fixed platform or on a slowly tilting platform (0.1 Hz sinusoid; 0.2 and 0.4 deg). Postural sway size was separated into two entities: (1) the spontaneous sway velocity component (natural random relatively rapid postural adjustments, RMS body angular velocity) and (2) the evoked tilt gain component (much slower 0.1 Hz synchronous tilt induced by the movement of the platform, measured as peak-to-peak (p-p) gain, ratio of body angle to applied footplate rotation).

**Results:**

There was no correlation between the velocity of an individual’s sway and their evoked tilt gain (*r* = 0.34, *p* = 0.15 and *r* = 0.30, *p* = 0.22). However, when considered separately, each of the two measurements showed fair to good absolute agreement within conditions. Spontaneous sway velocity consistently increased as participants were subjected to increasing disturbance. Participants who swayed more (or less) did so across all conditions [ICC_(3,k)_ = 0.95]. Evoked tilt gain also showed consistency between conditions [ICC_(3,k)_ = 0.79], but decreased from least to most disturbed conditions.

**Conclusion:**

The two measurements remain consistent between conditions. Consistency between conditions of two very distinct unrelated measurements reflects the idiosyncratic nature of postural sway. However, sway velocity and tilt gain are not related, which supports the idea that the short-term regulation of stability and the longer-term regulation of orientation are controlled by different processes.

## Introduction

Postural sway size in standing humans is different in distinct populations. It generally changes while we age, being higher in children and adolescents, lower in young adults and higher again in older adults (Sheldon, 1963; Goble and Baweja, 2018). Larger sway in children is associated with immature developmental processes (Nolan et al., 2005; Rival et al., 2005; Schedler et al., 2019), while in older adults, it reflects degeneration of neural and musculoskeletal systems (Horak et al., 1989), and can be indicative of larger incidence of falls (Piirtola and Era, 2006; Quijoux et al., 2020). Postural sway also tends to be greater in people with various disabilities compared to healthy individuals (Hufschmidt et al., 1980; Cameron and Lord, 2010; Deschamps et al., 2014). These studies have focused on postural sway size as a measure of group behavior. Unsurprisingly, postural sway size is often thought of as an indicator of instability although this is not necessarily so (for example Suzuki et al., 2020). Less attention has been directed at sway size as an individual characteristic. Does an individual’s sway size reflect his or her postural control mechanisms in the same way that the sway size of a group reflects the generalized properties of that group’s postural control mechanisms?

Intraclass correlation coefficient (ICC) is a measure of reliability that relates measurement error to the variability between persons. It can be subdivided in two main classes, tests of absolute agreement and tests of consistency (McGraw and Wong, 1996). The first is adopted when results should be as equal as possible, repeatedly, and systematic differences between results are not ignored. The latter is adopted when systematic differences between results are ignored. The results do not need to be equal, but it is important for the ranking of individuals to be consistent. Sway size is considered a reliable measurement of postural control mechanism because it has shown fair to excellent absolute agreement between trials in the same condition in quiet standing individuals (review by Ruhe et al., 2010). Sway size was also shown to remain in moderate agreement within individuals during large rotational perturbations mimicking falls (Dickstein and Dvir, 1993; Hill et al., 1995). In these studies, data were correlated from the same participant in the same condition at different times.

In the present study, we investigate if sway size is idiosyncratic. For sway size to be considered idiosyncratic, it should show good consistency when measured within the same participant in different conditions. The more discrepant the types of variables being tested for consistency, and the less the change in ranking of participants measured in various situations, the more idiosyncratic postural sway size should be. Yamamoto et al. (2015) analysed many different components of CoP variation in quiet standing and found that some were relatively common and others more idiosyncratic. However, to our knowledge, the consistency with which individuals’ COM moves in response to disturbances which provoke instability has not been investigated. That is, if one individual has a typical sway that is twice as big as another in normal standing, will the same difference pertain when both individuals are subjected to identical disturbances such as when standing in a moving vehicle or vessel? The question has obvious relevance to the liability of individuals to fall. In this study we address this issue.

To cover different aspects of sway, postural sway size is measured here as two different entities. The first is the relatively frequent spontaneous alterations in body velocity which are caused by intelligently controlled adjustments of neural drive to the calf muscles (Loram et al., 2005). The source of these adjustments does not concern us here. A second, less studied, aspect of postural sway in standing is the longer-term maintenance of an appropriate alignment with respect to gravitational vertical. When placed on a slowly tilting surface, standing subjects will adjust their mean position in sympathy with the applied tilt (Walsh, 1973; Gurfinkel et al., 1995; Osborne, 2013). Generally, adjustment of mean position is to a similar or even greater degree than the applied tilt. In this investigation we ask, first; whether spontaneous sway is consistent in individuals, second; whether tilting response is consistent in individuals, and third; whether an individual’s tendency to tilt is associated with his or her spontaneous sway. We test individuals in normal standing, and in conditions of subtle, slow sinusoidal antero-posterior tilts (small rotations of the standing platform about the ankle joint). We hypothesize that spontaneous sway and tilting tendency are concordant between trials and consistent between conditions, and that spontaneous sway and tilting tendency are unrelated.

## Materials and Methods

### Participants and Experimental Protocol

This analysis was performed using data obtained in the course of a previous experiment (Sakanaka et al., 2021), a study approved by the institution’s local human ethics committee (ERN_15-0674) and in conformity with the principles of the *Declaration of Helsinki*. It was conducted at the School of Sports, Exercise and Rehabilitation Sciences, University of Birmingham, Birmingham, United Kingdom. Data were collected from 19 healthy adults (eight female; age 29.2 ± 3.2 years; height 1.71 ± 0.1 m; weight 68.3 ± 11.7 kg; mean ± SD); all gave written informed consent.

Participants stood freely with eyes closed on footplates supported by a motorized platform (Copley Motion Systems, United Kingdom) and coaxially aligned with their ankles, a custom-made equipment already used in previous studies (Sakanaka et al., 2016, 2018). A light plywood board (1.2 m length, 0.5 m width, and total weight 1.2 kg) was strapped to the participant’s back with polyester straps at shoulder, waist and calf levels to reduce the participant’s use of hip and knee strategies for postural control. Anteroposterior body sway was recorded from a laser-reflex sensor (Model YT44MGV80; Wenglor, Germany) pointing directly at the board (sample rate 250 Hz).

Postural sway size was measured in three conditions aimed to increase levels of instability:

(1) *Normal*: locked footplates, horizontal position;
(2) *Sine 0.2*: footplates continuously tilting by a 0.1 Hz sine waveform of 0.2 deg peak-to-peak amplitude, horizontal mean position;
(3) *Sine 0.4*: footplates continuously tilting by a 0.1 Hz sine waveform of 0.4 deg peak-to-peak amplitude, horizontal mean position.

We expected that these three conditions, one stationary and two with increasing levels of platform tilt, would be associated with increased postural sway. Two trials of approximately 150 s were conducted for each of the three conditions (six trials in total, randomly assigned). A section of 90 s from each trial was used for analysis. Between each trial, participants were given approximately 1 min to rest. All trials were collected in 1 session of approximately 1 h.

### Data Analysis

#### Determination of Spontaneous Sway (RMS Body Angular Velocity)

Numerous descriptive measurements have been used to characterize body sway. There is currently no agreement of which parameter is the most appropriate (Visser et al., 2008; Ruhe et al., 2010; Scoppa et al., 2013). Sway velocity has been often adopted due to its satisfactory repeatability across trials and populations. It represents body velocity during naturally occurring body angle reversals (Geurts et al., 1993; Pai and Patton, 1997; Lafond et al., 2004), and has been associated with the amount of regulatory activity needed to obtain stability (Hufschmidt et al., 1980; Maki et al., 1991; Prieto et al., 1996). Accordingly, we measured sway velocity in this study.

**FIGURE 1:**
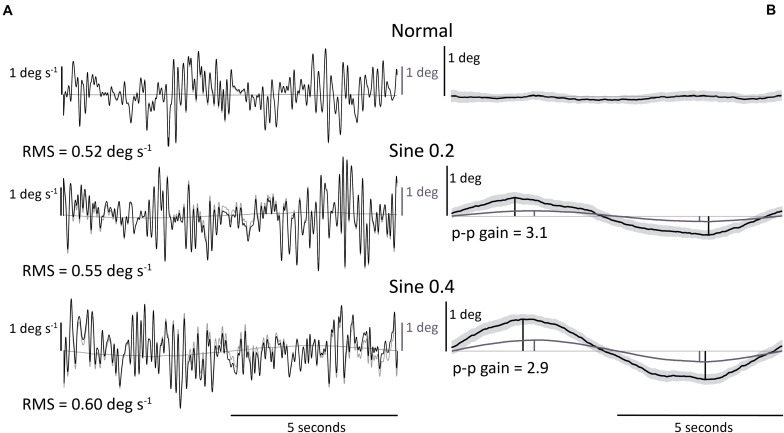
Illustrative representative dataset. **(A)** RMS body angular velocity. Section of body angular velocity from one participant in 3 conditions, with (black) or without (gray) 0.05–0.15 Hz notch Butterworth filter. Footplate angle (gray) is added for reference. **(B)** Body angle p-p gain (ratio of body to footplate angle). Average body angle from one participant in 3 conditions. Light gray area shows 95% confidence interval of data averaged across 30 trials. Footplate angle (gray) is added for reference. Body angle p-p gain was only calculated in disturbed conditions.

Body angle was calculated as the inverse tangent of the laser-reflex sensor data divided by the laser height above the ankles. This signal was filtered with a 10 Hz low-pass Butterworth filter to reduce the effect of noise (Raymakers et al., 2005; Duarte and Freitas, 2010), and with a notch filter at 0.05–0.15 Hz interval to remove the 0.1 Hz platform tilting component from the trials, and allow for the comparison between conditions of tilting versus horizontally locked platform. A Savitzky-Golay differentiating filter (polynomial order = 4, window length = 21, sample rate = 1000) was applied to body angle to estimate body angular velocity. Spontaneous sway velocity was calculated as the root-mean-square (RMS) of body angular velocity in normal, sine 0.2 and sine 0.4 conditions (Figure 1A).

An increased sway velocity can indicate increased amplitude of sway or increased frequency of sway. To clarify this, a spectral analysis was conducted. Power spectral density (PSD) was calculated with the Welch method (pwelch function from MATLAB, R2018a, Mathworks, MA, United States). Then PF50, calculated as the power frequency below which 50% of the total power is found (Prieto et al., 1996; Yamamoto et al., 2015), was estimated for the frequency range 0–2 Hz.

#### Determination of Evoked Tilt Gain (Body Angle p-p Gain)

Evoked tilt provoked by the tilting platform was measured with a time averaged sway size analysis. We averaged the body angle time-series data over the full 0.1 Hz sine-wave disturbance (nine cycles for each of the two trials) and measured its peak-to-peak amplitude (p-p) in sine 0.2 and sine 0.4 conditions. Peak-to-peak amplitude was measured as the distance between maximum and minimum rotations of the nine-cycle averaged body tilt as a ratio of footplate tilt. We expressed the size of this measurement of sway as a gain value (ratio p-p amplitude of body angle/p-p amplitude of platform angle) (Figure 1B).

#### Statistical Analysis

Statistical analysis was performed with RStudio: Integrated Development for R (Version 1.1.453, RStudio Team, 2016, RStudio, Inc., Boston, MA, United States). Two-way repeated measures ANOVA was used to compare mean differences of RMS body angular velocity and body angle p-p gain in different trials (#1 and #2) and conditions (normal, sine 0.2, and sine 0.4). A Greenhouse-Geisser correction was used for the main effect of condition because Mauchly’s Test of Sphericity indicated that the assumption of sphericity had been violated. Intraclass correlation coefficients and their 95% confidence intervals were calculated based on: (1) single- (ICC_(1,1)_) and (2) mean-ratings (ICC_(1,k)_), absolute agreement, one-way random-effects ANOVA model, and (3) mean-rating (ICC_(3,k)_), consistency, two-way mixed-effects ANOVA model (Shrout and Fleiss, 1979; McGraw and Wong, 1996; Koo and Li, 2016). The first was used to check within-subject variability between trials of spontaneous sway velocity, the second was used to check within-subject variability between average of nine cycles within each trial of evoked tilt gain (both measures of absolute agreement), and the third was used to check mean between-condition variability (measure of consistency). ICC < 0.40 results were considered poor, 0.40 ≥ ICC < 0.75, fair, 0.75 ≥ ICC < 0.90, good, and ICC ≥ 0.9, excellent (Rosner, 2015). Pearson’s correlation coefficient (*r*) was used to measure the strength of the association between RMS body angular velocity and p-p gain in body angle. *p* < 0.05 was considered statistically significant for all tests.

## Results

### Sway (RMS Body Angular Velocity) Increases With Increasing Disturbance, and Is Concordant Between Trials and Consistent Between Conditions

**FIGURE 2:**
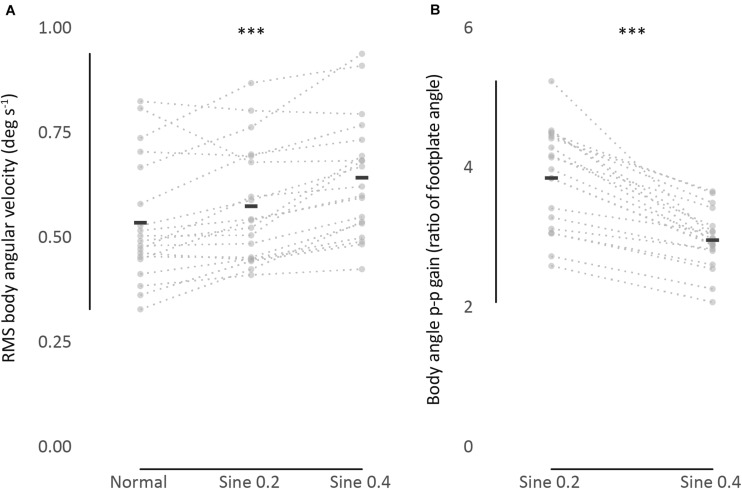
**(A)** RMS body angular velocity. **(B)** Body angle p-p gain (ratio of body to footplate angle). Univariate scatter plot for different standing conditions, average from 2 trials (gray dots). Black bars indicate mean values. Dotted lines connect data from each participant. ^∗∗∗^*P* < 0.001.

These results were relevant to normal, sine 0.2, and sine 0.4 conditions. A wide range of RMS body angular velocity was found between participants and conditions (min = 0.32, max = 1.02 deg s^–1^, one participant in normal and another participant in sine 0.4 conditions, respectively). On average, mean sway velocity of all participants increased from normal to sine 0.2 to sine 0.4 conditions (0.54 → 0.58 → 0.65 deg s^–1^, Figure 2A and Table 1).

**TABLE 1:** Descriptive data.

Variable	Condition	Trial#1 (Mean ± SD)	Trial#2 (Mean ± SD)	Average (Mean ± SD)	Range from averages (min–max)
RMS body angular velocity (deg s^–1^)	Normal	0.51 ± 0.15	0.57 ± 0.16	0.54 ± 0.15	0.33–0.83
	Sine 0.2	0.58 ± 0.14	0.58 ± 0.15	0.58 ± 0.14	0.41–0.87
	Sine 0.4	0.65 ± 0.16	0.64 ± 0.14	0.65 ± 0.14	0.43–0.94
Body angle p-p gain (ratio of body to footplate angle)	Sine 0.2	3.74 ± 0.79	4.00 ± 0.83	3.87 ± 0.72	2.61–5.25
	Sine 0.4	3.02 ± 0.45	2.94 ± 0.47	2.98 ± 0.42	2.09–3.68

This mean difference between conditions was significant, as shown by a two-way repeated measures ANOVA analysis, *F*(1.4, 25.9) = 21.36, *p* < 0.001. *Post hoc* Bonferroni test showed that sway in sine 0.4 condition was higher than in sine 0.2 and normal conditions, and sway in sine 0.2 condition was higher than in normal condition. As expected, no significant effect of trial number was found, *F*(1,18) = 3.46, *p* = 0.079, and the interaction between condition and trial was also not significant, *F*(2,36) = 3.03, *p* = 0.061.

PF50 analysis showed no significant difference between conditions [0.65 ± 0.19 Hz in normal, 0.59 ± 0.11 Hz in sine 0.2, and 0.64 ± 0.12 Hz in sine 0.4 condition, *F*(2,36) = 2.09, *p* = 0.138], and no correlation between PF50 and RMS body angular velocity was found (*r* = −0.14, *p* = 0.57 in normal, *r* = −0.02, *p* = 0.92 in sine 0.2, and *r* = 0.20, *p* = 0.42 in sine 0.4 condition).

Next, the consistency of the estimates was investigated. First, we checked if absolute agreement was found between two trials of 90 s each. We found fair to good agreement in normal condition [ICC_(1,1)_ 95% CI 0.43–0.88], and fair to excellent agreement in disturbed conditions [ICC_(1,1)_ 95% CI 0.57–0.96], showing that RMS body angular velocity is an agreeable measurement between trials within conditions, whether the participant was standing quietly or being disturbed. Second, we verified consistency between conditions from averaged trial results. We found excellent consistency [ICC_(3,k)_ 95% CI 0.90–0.98], showing that participants who swayed less when quietly standing consistently swayed less while being disturbed (Table 2). A visual representation of the consistency between conditions can also be seen when we rank participants and compare ranking between conditions (Table 3, columns 1–4). When we further divide the ranking results in groups of 4–5 participants and shade them differently, the gray shading shows how sway size remains relatively consistent between conditions.

**TABLE 2:** Intraclass Correlation Coefficient (ICC) results.

Variable	Condition	ICC_(1,1)_ ρ (95% CI) between trials	F test with true value 0			
			Value	df1	df2	*p*-Value
RMS body angular velocity	Normal	0.73 (0.43–0.88)	6.3	18	19	0.000
	Sine 0.2	0.89 (0.74–0.96)	17.0	18	19	0.000
	Sine 0.4	0.81 (0.57–0.92)	9.4	18	19	0.000
Single-rating, one-way random effects, absolute agreement.						

		**ICC_(1,k)_ ρ (95% CI) between trials, k = 9**				

Body angle p-p gain	Sine 0.2	0.73 (0.30–0.89)	3.7	18	19	0.004
	Sine 0.4	0.79 (0.46–0.92)	4.7	18	19	0.001
Multiple-rating, one-way random effects, absolute agreement.						

		**ICC_(3,k)_ ρ (95% CI) between conditions, k = 2**				

RMS body angular velocity		0.95 (0.90–0.98)	21.0	18	36	0.000
Body angle p-p gain		0.79 (0.44–0.92)	4.7	18	18	0.001
Multiple-rating, two-way mixed effects, consistency. df, degrees of freedom; CI, confidence interval.						

**TABLE 3:** Ranking in increasing order of RMS body angular velocity and body angle p-p gain (ratio of body to footplate angle) from averaged results of 2 trials.

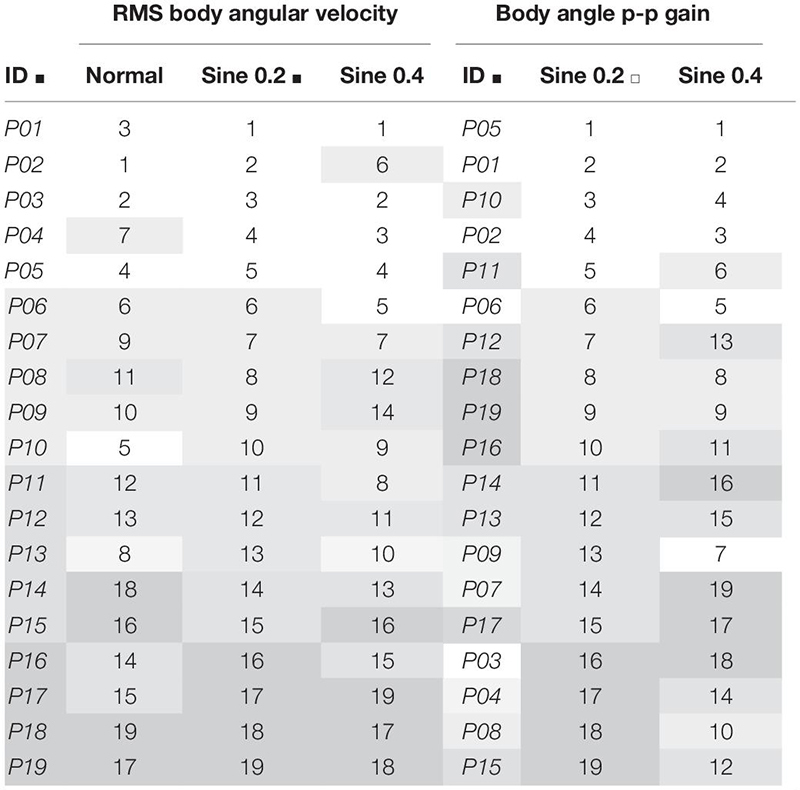

**FIGURE 3:**
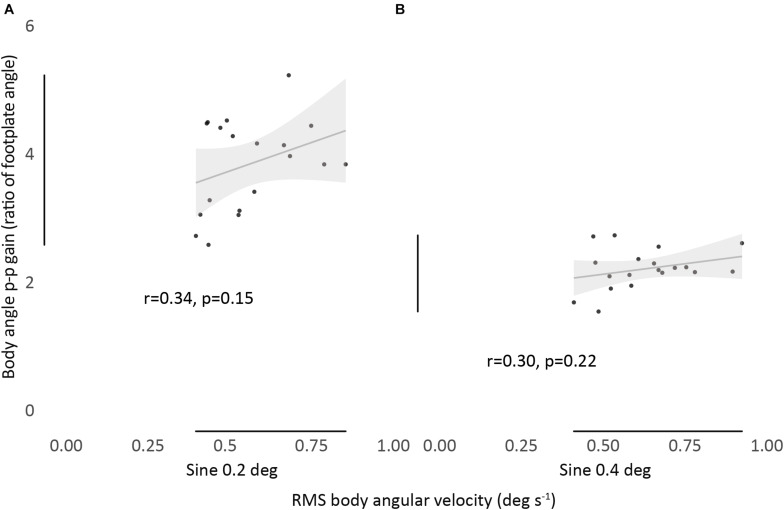
Relationship between RMS body angular velocity and body angle p-p gain in sine 0.2 **(A)** and sine 0.4 **(B)** conditions. Bivariate scatter plot with regression line and confidence interval band (95% CI). Pearson’s r and *P*-values show a non-significant correlation between all conditions.

*For each measurement, values were ordered and ranked in relation to sine 0.2 condition (columns 3 ■ and 6 □, respectively). Participant ID (ID columns 1 ■ and 5 ■) was defined based on sine 0.2 condition RMS body angular velocity ranking. Participants were further divided in groups of 4–5, each group represented by different shades of gray. Lighter shades represent lower values. P03 ranked 3rd in RMS body angular velocity in sine 0.2 condition and ranked 2nd in normal and sine 0.4 conditions. The same P03 ranked 16th and 18th in body angle p-p gain in sine 0.2 and sine 0.4 conditions. Order of participants (ID columns 1 ■ and 5 ■) varied between two entities due to non-significant correlation between them (Figure 3A).*

### Evoked Tilt Gain (Body Angle p-p Gain) Decreases With Increasing Disturbance, and Is Concordant Between Trials and Consistent Between Conditions

These results were relevant to sine 0.2 and sine 0.4 conditions. Once again, there was a large range of values of gain between participants. In an absolute sense the larger tilt of the platform (0.4 deg) produced a larger tilt of the participant (Figure 1B). However, when expressed as a gain (ratio of participant tilt to platform tilt), the gain was less for the larger tilt (3.87 → 2.98 p-p gain, Figure 2B and Table 1), *F*(1,18) = 61.05, *p* < 0.001. No significant effect of trial number, *F*(1,18) = 0.92, *p* = 0.349, and no significant interaction between condition and trial was found, *F*(1,18) = 3.02, *p* = 0.099.

Absolute agreement between trials was poor to good in sine 0.2 condition [ICC_(1,k)_ 95% CI 0.30–0.89], and fair to excellent in sine 0.4 condition [ICC_(1,k)_ 95% CI 0.46–0.92]. Consistency between conditions was fair to excellent [ICC_(3,k)_ 95% CI 0.44–0.92], showing that participants were consistently tilting proportionally less with increasing disturbance (Table 2). From ranking analysis, we can again visualize the consistency between conditions when comparing grading of gray between groups (Table 3, columns 5–7).

### There Is No Correlation Between Spontaneous Sway (RMS Body Angular Velocity) and Evoked Tilt Gain

We found no significant correlation between the magnitude of RMS body angular velocity and body angle p-p gain, for any condition, showing no association between measurements (Figure 3).

## Discussion

Postural sway consists of an apparently random fluctuation in body angle occurring when individuals stand upright. In this experiment, when the standing platform was oscillated slowly (0.1 Hz) the mean velocity of this spontaneous sway increased slightly. The imposed slow oscillation was also clearly superimposed on the position of the COM. This tilting response was measured as the ratio of body angle to platform angle. We hypothesized that sway and tilt characteristics in standing healthy adults would be concordant within and consistent between conditions. This hypothesis was broadly confirmed. Individual spontaneous sway velocity was repeatable within conditions and consistently increased from normal to during tilting. Individual evoked tilt gain was repeatable within conditions and consistently decreased with increasing disturbance. Confirming our hypothesis, we found no clear relationship between the tendency of individuals to sway and their propensity to tilt.

### The Standing Process—Sway and Tilt

The standing human body attains stability while maintaining the vertical projection of the body COM within the stability limits determined by the base of support (BOS). In accomplishing this feat, the postural muscles provide torque which counteracts the toppling tendency of the body that is the result of gravity. Normally, the antero-posterior torque is provided mainly by the ankle plantarflexors, and this is explicitly the case in the present experiments where the body is splinted by a rigid back support, minimizing any contribution from hip or trunk muscles. Numerous experiments involving balancing artificial inverted pendulums (both real and virtual) and splinted human subjects, have shown a similarity in the balancing process (Fitzpatrick et al., 1992, 1994; Loram and Lakie, 2002; Lakie et al., 2003; Lakie and Loram, 2006; Loram et al., 2011; Morasso et al., 2019). The advantage of the present approach is that it removes uncertainty about intersegmental movement and produces an unambiguous COM. The disadvantage is that the sway size may be larger than in normal standing. One important role of the standing process is to stabilize the otherwise unstable body. Any such balancing process will result in a mean position of the COM over time, and it is a second role of the standing process to ensure that this mean position remains appropriate. For most people the body is inclined slightly forward so that the mean vertical projection of the COM is on average a few centimeters forward of the ankle joint. There is uncertainty about the source of the “kinaesthetic reference” which presumably defines this goal in standing (Gurfinkel et al., 1995). Normally vision plays an important role, but this is excluded in the present experiments. A reference to gravitational vertical signaled by the otolith organs would seem a possibility, but previous (Walsh, 1973; Gurfinkel et al., 1995) and the present experiments have shown that when the supporting surface is tilted the kinaesthetic reference moves with it, so that the mean position of the body tilts slowly with the supporting surface. Therefore the goal is not determined by a gravitational frame of reference. Gurfinkel et al. (1995) reported that there was a considerable lag between platform tilt and body tilt. Taken together, these observations strongly suggest that proprioceptive sources of information are of most importance.

### The Effects of Tilting—Tilt Gain

In our experiments the main source of the kinaesthetic reference is the support surface. The perturbations were small and slow, and participants were unable to reliably report that the platform was moving or that they felt that they were being destabilized. Nevertheless, all participants were affected by platform rotation and moved in a way that exaggerated the platform tilt, particularly striking in sine 0.2 deg condition when they tilted nearly four times as much as the platform. Apparently, the small spontaneous sways become biased so that their reversals do not entirely cancel, accumulating over the duration of the platform tilt, so that the participant tilts more than the platform. Similar exaggerated responses to unperceived tilt were recorded by Walsh (1973). Gurfinkel et al. (1995) used larger (1.5 deg) and much slower (∼150 s p-p) sinusoidal tilts and their participants tilted 0.41–1.72 as much as the platform. For very large perturbations, proprioceptive perception of change in ankle angle should be much clearer. Presumably in this case, the risk of falling becomes apparent and evoked tilt gain reduces so the person tilts less than the platform. There is a stability limit to the amount of sway that people can handle (Horak et al., 1989; Riach and Starkes, 1993), and COM amplitude will not increase proportionally as perturbation size progressively increases (Peterka, 2002; Cenciarini and Peterka, 2006). The disturbances in our study were subtle and did not drive participants towards their stability limit. As the size of the tilting is increased, and perception of platform angle improves, evoked tilt gain reduces (sine 0.4 condition, Figure 2B). The novelty of the present results is the observation that people have idiosyncratic responses to tilt, responding to tilt in a similar way in repeated trials (Table 2).

### The Effects of Tilting—Mean Sway Velocity

Tilting provoked a small but highly significant systematic increase in individuals’ mean sway velocity. Consequently, an individual with a normally small sway velocity would sway more when disturbed, but this increased sway velocity might well be smaller than that of another individual in undisturbed conditions (Figure 2A). An increase in sway velocity can indicate an increase in sway amplitude or an increase in the frequency of the sway. There was no significant tendency for the participants with the largest sway velocities to have the highest sway frequencies, so we suggest that our measurements reflect their mean sway amplitude. Furthermore, PF50 analysis shows that there is no significant rise in frequency during tilting, so we suggest that it is the mean amplitude of the sway that increases. Sway amplitude, which expresses the amount that an individual wobbles, is often intuitively associated with instability although this is not necessarily true (Suzuki et al., 2020).

Because they observed frequently repeated adjustments in COP during tilting, Gurfinkel et al. (1995) suggested that the balancing process continues as normal during slow tilting though not around a fixed set-point but relative to a slowly and continuously changing setpoint. The present results support this observation and also suggest that the balancing process is associated with faster swaying during tilting. The increased velocity of the standing sways is not simply the direct additive effect of the platform tilt because all velocity records were notch filtered to remove the imposed tilting velocities. The mean sway velocity (∼0.5 deg s^–1^; Figure 2A) is at least an order of magnitude greater than the tilting velocity (0.04 deg s^–1^ max).

Previous studies obtained fair to excellent agreement for sway size between trials in the same condition in standing individuals (Ruhe et al., 2010). One study with perturbed conditions (Hill et al., 1995) obtained ICC_(2,1)_ = 0.55 (quiet) → 0.82 (sinusoidal 8 deg amplitude rotation in 4.15 s) → 0.89 (same amplitude in 8.3 s condition). In our study, we obtained ICC_(1,1)_ between 0.73 and 0.89. The results show that, regardless of standing condition and perturbation type and intensity, ICC remains within the range fair to good. Although the velocity of an individual’s sway under normal standing conditions predicts how much they will sway when disturbed it does not predict how much they will tilt (Figure 3). There is little published work on this issue. Maki et al. (1990) also found no relationship between spontaneous sway (speed of COP displacement) and induced sway (saturation amplitude, a predictive measurement of the amount of platform translation necessary to obtain maximum COP displacement) when testing different populations. However, these were much larger responses than the modest tilts we induced.

### Consistency of Spontaneous Sway Velocity and Evoked Tilt Gain Reflects the Idiosyncratic Nature of Postural Sway Size

The studies mentioned above focused on *within*-condition absolute agreement, not consistency *between* conditions. To characterize sway as idiosyncratic, we performed a between-condition analysis. ICC between spontaneous sway in different conditions was excellent [ICC_(3,k)_ = 0.95], and good for evoked tilt [ICC_(3,k)_ = 0.79]. This idiosyncratic nature of sway is clearly seen in Figure 2, as dotted lines connecting each participant data point from different conditions show a clear level of parallelism, both in sway velocity and in tilt gain. Similarities in ranking distribution further confirm this consistency (Table 3). Consistency between conditions is much higher, though, in spontaneous sway velocity than in evoked tilt gain. This might be related to the non-linear range changes in evoked tilt gain.

### The Relationship Between Tilt Gain and Mean Sway Velocity

Our results strongly support the idea that the process responsible for spontaneous sway in standing, is distinct in some way from the process which sets the kinaesthetic reference. The stereotyped response of our participants to tilting suggests that individuals have a typical standing position which shifts to a greater or lesser extent in a characteristic way when they are tilted. However, their ability to preserve their goal is not related to their ability to minimize their sway velocity. Subjects have a characteristic sway velocity and this is increased to a moderately predictable degree by disturbance. Presumably their sway velocity is a reflection of mechanical factors such as their intrinsic ankle stiffness and the adequacy of their neural stabilization process. Conversely, the ability to generate a reference position requires integration over a considerable period and the synthesis of signals from various sources. However, this similarly stereotyped and relatively predictable ability bears no obvious relationship to their ability to control their sway velocity, so it is presumably a reflection of different, probably higher level, neural processing.

### Study Limitations and Implications

Our analysis was confined to anteroposterior sway of a splinted body in participants with eyes closed. Accordingly the results should be applied with caution to people standing normally. When freely standing, all the joints of the body are mobile (Day et al., 1993; Hsu et al., 2007), and body sections can compensate for the sway of different body sections. This, and the unfamiliarity of standing splinted, probably explains why splinted individuals sway rather more than normal (Fitzpatrick et al., 1994; Loram and Lakie, 2002). Preservation of standing position is much improved when vision is permitted, and sway size is also decreased. With these limitations our results do suggest that individuals who sway more when on a stationary surface will have larger sway when that surface is non-stationary. However, it may be that it is more exaggerated responses to tilt which better predict individuals who are at particular risk of falling when the supporting surface is moved, and these will not necessarily be individuals with large sway.

## Conclusion

There was no correlation between individuals’ sway velocity and their propensity to tilt, supporting the idea that the short-term regulation of stability and the longer-term regulation of orientation are controlled by different processes. Furthermore, both measurements were shown to be consistent between varying standing conditions. Consistency between conditions of two very distinct measurements of postural sway size reflects its idiosyncratic characteristic.

## Data Availability Statement

The datasets presented in this study can be found in online repositories. The names of the repository/repositories and accession number(s) can be found in the article/Supplementary Material.

## Ethics Statement

The studies involving human participants were reviewed and approved by the Human Ethics Committee at the School of Sports, Exercise and Rehabilitation Sciences, University of Birmingham, Birmingham, UK (ERN_15-0674). The patients/participants provided their written informed consent to participate in this study.

## Author Contributions

TS co-planned the study design, recruited participants, performed data collection, processing and analysis, contributed to data interpretation, manuscript preparation, and revisions. RR co-planned the study design and made the final decision to submit the manuscript for publication. ML contributed to data interpretation, manuscript preparation, and revisions. All authors contributed to the article and approved the submitted version.

## Conflict of Interest

The authors declare that the research was conducted in the absence of any commercial or financial relationships that could be construed as a potential conflict of interest.

## Abbreviations

RMS: root mean square
p-p: peak-to-peak.
